# e-MIR^2^: a public online inventory of medical informatics resources

**DOI:** 10.1186/1472-6947-12-82

**Published:** 2012-08-02

**Authors:** Guillermo de la Calle, Miguel García-Remesal, Nelida Nkumu-Mbomio, Casimir Kulikowski, Victor Maojo

**Affiliations:** 1 Biomedical Informatics Group, Departamento de Inteligencia Artificial, Facultad de Informática, Universidad Politécnica de Madrid, Boadilla del Monte, 28660 Madrid, Spain; 2 Department of Computer Science, Rutgers – The State University of New Jersey, New jersey 08855, USA

**Keywords:** Medical informatics, Cataloging, Classification, Software resources, Information storage and retrieval, Search engine, Database, Information management, Folksonomies, Social tagging

## Abstract

**Background:**

Over the past years, the number of available informatics resources in medicine has grown exponentially. While specific inventories of such resources have already begun to be developed for Bioinformatics (BI), comparable inventories are as yet not available for the Medical Informatics (MI) field, so that locating and accessing them currently remains a difficult and time-consuming task.

**Description:**

We have created a repository of MI resources from the scientific literature, providing free access to its contents through a web-based service. We define informatics resources as all those elements that constitute, serve to define or are used by informatics systems, ranging from architectures or development methodologies to terminologies, vocabularies, databases or tools. Relevant information describing the resources is automatically extracted from manuscripts published in top-ranked MI journals. We used a pattern matching approach to detect the resources’ names and their main features. Detected resources are classified according to three different criteria: functionality, resource type and domain. To facilitate these tasks, we have built three different classification schemas by following a novel approach based on folksonomies and social tagging. We adopted the terminology most frequently used by MI researchers in their publications to create the concepts and hierarchical relationships belonging to the classification schemas. The classification algorithm identifies the categories associated with resources and annotates them accordingly. The database is then populated with this data after manual curation and validation.

**Conclusions:**

We have created an online repository of MI resources to assist researchers in locating and accessing the most suitable resources to perform specific tasks. The database contains 609 resources at the time of writing and is available at http://www.gib.fi.upm.es/eMIR2. We are continuing to expand the number of available resources by taking into account further publications as well as suggestions from users and resource developers.

## Background

Over the past years, thousands of very diverse biomedical resources have been made available over the Internet. To manage this wide range of resources, novel management approaches have been proposed [1-3]. To face this new situation, bioinformatics professionals have made significant efforts towards identifying and classifying resources [4-7]. The creation, maintenance and updating processes of repositories of resources are time-consuming tasks since they are usually manually performed. Recently, however, novel automatic or semi-automatic methodologies are being proposed to facilitate the management of the growing number of bioinformatics resources [3].

In contrast, few efforts have been devoted to retrieve and organize existing Medical Informatics(MI) resources. Currently, while MI databases and tools are growing exponentially there do not yet exist, to our knowledge, broadly accepted and regularly updated catalogues of MI resources. It is expected that the number of MI databases and tools continue will growing in the coming years [8]. Many professionals might benefit from using catalogues of MI resources, accessing open resources that are available over the Web.

In this paper we report on the design and implementation of the electronic-Medical Informatics Repository of Resources (e-MIR^2^) system, a free web-based application to discover, search and locate existing MI resources reported in the MI literature. We have especially focused on the identification of open source resources. For this purpose, we have adapted and expanded our prior research in bioinformatics, based on a custom pattern-matching approach [4,9-11].

## Construction and content

A range of MI resources reported in the literature have been classified using information automatically extracted from texts. This classification was carried out according to three different categorizations: functionality, type of resource and domain. Functionality describes the intended use for the resource—e.g. analyze, store or search—, type denotes the type of software resource—e.g. database, web service, inventory, etc.—and domain specifies the concrete area of application of the resource—e.g. imaging, eHealth, nursing, etc. We have developed different classification schemas for each of these categories.

### Developing the classification schemas

First, we searched for existing taxonomies used in the MI domain to classify informatics resources [12-15].We did not find any suitable taxonomy for the MI field, since most of the existing resource taxonomies were focused on Bioinformatics (BI) [4,16,17]. These taxonomies were not appropriate to index the much broader field of MI resources. Instead, we decided to build three different classification schemas for resource classification, following a systematic approach based on the idea of folksonomies [18]. Folksonomies became popular in the framework of the Web 2.0 to annotate information or images [19]. A key aspect of folksonomies is that tags are not imposed by a strict classification given by experts - rather, they are selected depending on how people use them in a specific domain. In our case, we decided to define our classification schemas using the idea of folksonomy tags, i.e. to define the categories selecting only concepts used by MI professionals to describe informatics resources.

To create the different classification schemas, we defined a training set of 204 papers containing information about resources from the literature. These papers were manually selected from top-ranked MI journals by a panel of experts with different backgrounds in medical informatics, bioinformatics, nano informatics and medical imaging. These journals were: International Journal of Medical Informatics (IJMI), Journal of the American Medical Informatics Association (JAMIA) and the Journal of the Medical Internet Research (JMIR). Titles and abstracts of the selected manuscripts were automatically processed to extract all terms in the text using a simple lexical analyzer developed with Python 3.6 [20]. Stop words were automatically discarded using a stop word filter provided by the NLTK utilities [21]. We decided to use the titles and abstracts of the manuscripts instead of the full text since they are publicly available and often contain the most representative and descriptive information regarding the resources. This information includes, for instance, names of resources, resource types, resource functionalities and/or whether resources are open source or not.

**Figure 1:**
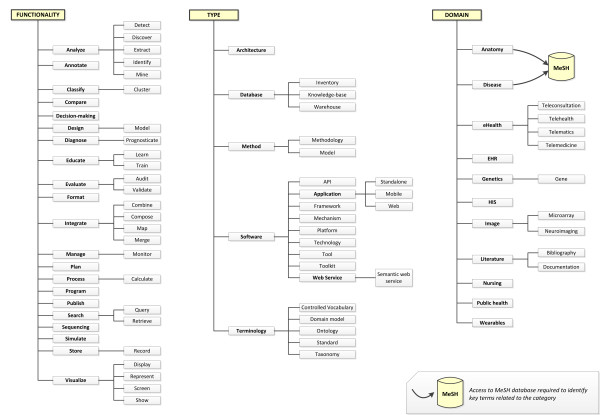
**e-MIR**^**2**^**classification schemas.** Medical informatics resources are classified in the e-MIR^2^ system according to three different categories: functionality, type of resource and domain. Each category is composed of several subcategories. Resources can be tagged with one or several concepts from the classification schemas.

To extract the most representative words in the manuscripts foreach classification schema, we focused on specific terms. For instance, in the functionality classification schema, we only considered the verbs, since verbs are most often used in research papers to describe the intended functionality of a resource. All the verbs were stemmed—i.e. reduced to its lemma or base form—using an open source implementation of the widely used Porter Stemmer [22]. Then, the resulting lemmas were ranked in descending order according to their frequency. The same panel of experts mentioned above manually reviewed the obtained rank, discarding terms or lemmas that were not relevant to MI. The selected verbs were then manually grouped into different clusters. From each cluster, one verb was selected to be the representative of the cluster. This verb was promoted to a higher level, with the rest of verbs appearing as its children in the hierarchy. This process was repeated with all clusters until the classification schema is completed. Synonyms were also considered and included in the classification schema. The classification schema we created for resource functionalities is shown in Figure 1.We used the same approach to create the resource type and the domain classification schemas, but in both cases we considered common nouns instead of verbs. The final classification schemas we obtained for resource types and domains are also illustrated in Figure 1.

### Description of the database

The information stored in the database describing resources is structured as follows:

· *Resource name*

· Manuscript information

· *Title*

· *Abstract*

· *Origin source*

· *Paper ID*

· *Path* or *URL*, to retrieve the paper’s information

· *XML record*

· *Open Source*, which indicates whether a resource is open source or uses open source technologies

· *Links*, detected within the paper

### Extraction of essential information

We created an automated text analyzer that scans the titles and abstracts of PubMed manuscripts to extract the essential information—i.e. name, links and open source—described in the previous section. We did not use the full text of the articles since it is not available for many manuscripts indexed by PubMed. We used simple pattern-matching techniques based on regular expressions to extract the names of the different resources reported in the manuscripts. We detected the names of resources focusing on the titles of the manuscripts. A set of patterns was created by the experts according to the most frequent or common textual patterns manually discovered in different manuscripts. An example of regular expression for discovering resource names is the following:

(1) [ˆ()*[a−z0−9]*[A−Z][a−zA−Z0−9]*((|−)[a−z0−9]*[A−Z][a−zA−Z0−9]*)*:

This pattern matches resource names which appear at the beginning of the title and are delimited by a colon. For instance, the title “3D-VIEWER: an atlas-based system for individual and statistical investigations of the human brain” matches this pattern, thus obtaining “3D-VIEWER” as the resource name.

If the text analyzer fails to detect a resource name, the manuscript is set aside by the algorithm. Otherwise, the manuscript is further processed to extract additional information such as, for example, links to web pages containing the resource or whether it is open source or not. This information is also extracted using regular expressions and stored in the database. The current availability of extracted links is periodically checked by the system. Additionally, the algorithm stores the complete information about the manuscript retrieved from the sources, i.e. title, abstract, origin source—e.g. PubMed—, manuscript ID at the origin source, path or URL to the origin source and an XML record containing the complete information about the paper in XML format. The manuscript ID and the path allows tracing to the original location where the resource information is available.

### Resource annotation and classification

We have also built a resource annotator that classifies the discovered resources into one or more categories belonging to the classification schemas described above. A lexical analyzer parses each manuscript title and abstract searching for mention of words associated with concepts in the different classification schemas. Words extracted from the manuscript’s title and abstract by the text analyzer are reduced to their lemma form and then matched against terms (lemmas) associated with the different categories. If a match is found, then the resource is annotated with the corresponding category in the classification schema. Such annotations are used later to filter the queries launched by users.

The annotation algorithm behaves differently for two specific categories in the domain classification schema: anatomy and diseases. The algorithm considers the entire branch of MeSH terms [12] devoted to such topics, searching the manuscripts for occurrences of diseases or parts of the body. Once a match is found, the resource is annotated with the tag “disease” or “anatomy” respectively instead of using the specific MeSH term. We made this choice to improve usability and to reuse well-established existing terminologies - in this case the MeSH terms.

Additional rules and constraints were used for the annotation of resources: i) any resource may belong to one, or more than one category within the same classification schema, and ii) while it is not necessary that all resources have matches in all classification schemas, every resource must belong to at least one of the categories of the classification schemas.

### Populating the database

To populate the e-MIR^2^ database, we assessed the possibility of using different subsets of MI journals but finally decided to consider all journals (22) included in the Journal Citation Report (JCR) index within the MI category provided by the ISI Web of Knowledge [23]. We retrieved from PubMed all the manuscript records belonging to the journals published before May 8^th^, 2012, using services provided by BioPython utilities [24]. The total number of papers retrieved and considered by our algorithm was 37996. During the extraction process, the algorithm identified 764 potential resources. Both duplicates and non-classified resources were automatically discarded by the algorithm. Resources were considered as duplicates when they were mentioned in more than one article, in the same or a different journal. The algorithm detected 47 duplicates, and 48 resources were not classified according to any classification schema. Then, the database was manually curated by the panel of experts to identify inconsistencies or misclassified resources. Nine of the 48 non-classified resources were identified as actual resources, while69 resources were discarded from the database (false positives). Finally, the database was populated with information associated with609 different resources.

Additionally, the algorithm extracted 31 links related to the resources from 26 different papers. No additional links were detected in the remainder of the manuscripts. Finally, the system labeled 24resources as open source resources or resources using open source technologies.

### Database curation

Curation of the database content was done manually using a web application. Two user profiles have been defined in the system: reviewers and administrators. The application provides a private and controlled area for these users. Both reviewers and administrators require a username and password to access the private zone.

The administrator profile has been designed to set up and administer the application, including features needed for these roles. The reviewer profile is defined to make it easier for experts to curate the database. Through the web interface, the curation process can be carried out by any authorized expert remotely, using a web browser with access to the Internet.

Our system also has a mechanism to allow users to become involved in database curation. While users cannot directly modify the content of the database, they can send an email using the link provided in the header of the main page. Comments received by email are evaluated both by the administrator of the system and at least one expert reviewer. If the evaluation result is positive, changes are incorporated into the database. This method of sending comments can also be used to suggest other improvements to the system.

### Updating the database

The e-MIR^2^ system has been designed to automatically update the database contents using the same method that helped build the initial database. To carry out the update process, the application retrieves from PubMed new manuscripts published since the last update. The information about the new manuscripts is processed using the algorithm described above. New detected resources are then included in the database and labeled as *pending approval* until a reviewer has checked them.

In addition, e-MIR^2^ provides a page that allows users to suggest new resources not included in the system. The link to this page is located in the main page header and labeled as *send a suggestion*. This page is used to collect information about the user—basically her name and email—and a new resource(s) suggested. Users can indicate the resource name, whether the resource is open source or not, a paper title—if the resource has been published in a scientific journal—, resource description, abstract and relevant links. The user is also allowed to manually classify the resource using our three classification schemas. The user must specify at least one category from one of the three classification schemas. Once the form has been reviewed and validated, the information is stored in the database and tagged as *pending approval*. After the reviewers evaluate the new resource, an email is sent to the user notifying her of the decision. If it is an acceptance, a direct link to the resource is provided in the notification email. If the suggested resource is already registered in the database, then the user is notified about this, and is then linked to the page describing the resource.

## Utility

**Figure 2:**
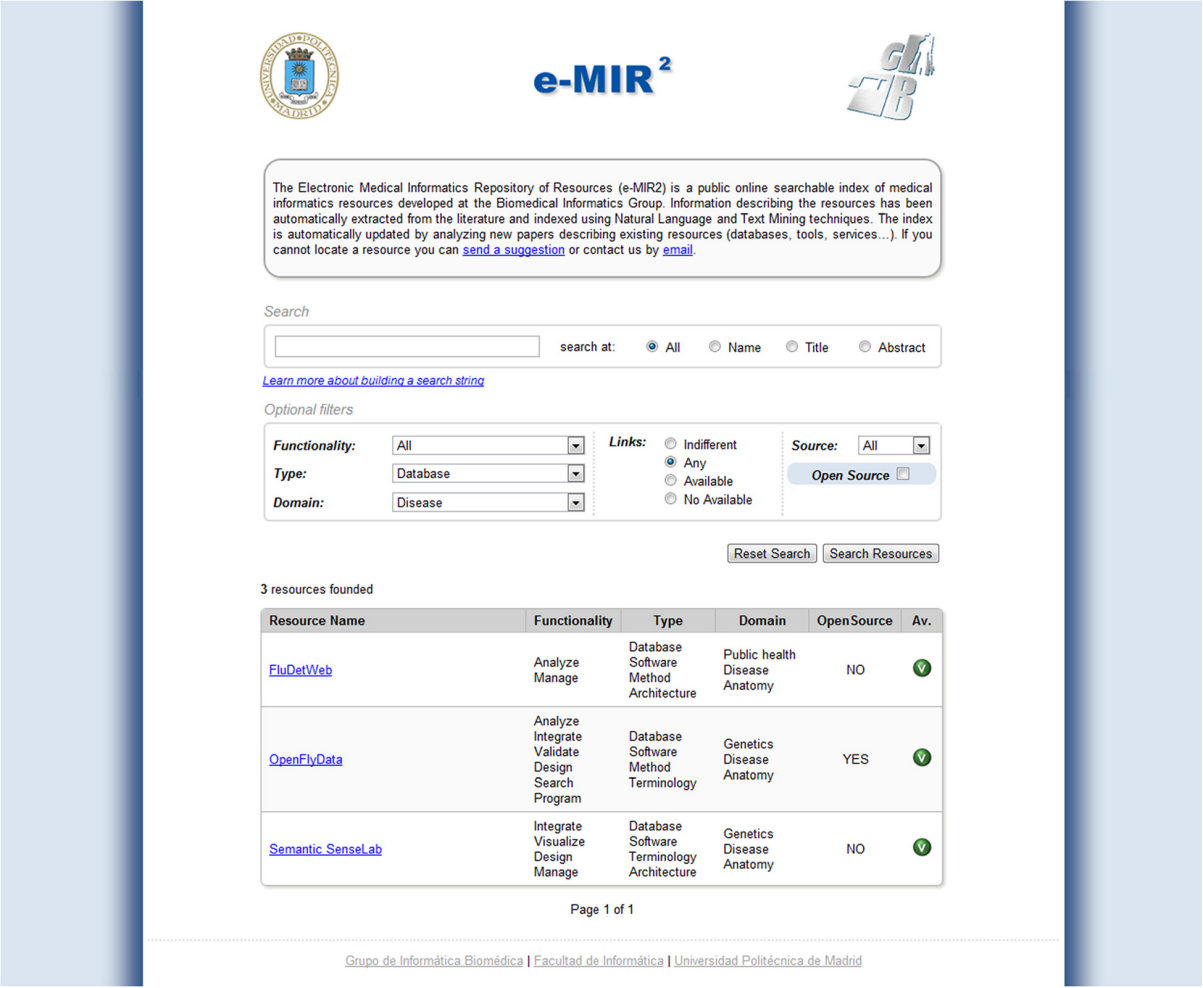
**Screenshot of the e-MIR**^**2**^**web application.** The e-MIR^2^ web application allows users to search for Medical Informatics resources by specifying a search string. To refine searches, users can apply different optional filters related to functionalities, type of resources, domains, links or open source. Search results are displayed on the same page in a tabular format.

We developed a web application to access the contents of the database of MI resources. This online application is freely available at http://www.gib.fi.upm.es/eMIR2. No registration is required to access the system. Figure 2 shows a screenshot of the e-MIR^2^ application. The application has been optimized for Mozilla Firefox® and Google Chrome®.

To search for a specific resource, the application asks users to fill in a text box specifying the search string—e.g. the name or part of the name of the resource. The search string can be composed using regular expressions and the logical operators AND and OR. Usage instructions and examples are provided following the link below the search text box. Additionally, several optional filters are provided to refine the search results. Filters can be applied over different fields, such as the search string, functionality, type of resource, domain, links, publication source and open source. By default, the scope for the search string is not limited—i.e. the application searches the complete database—but users can focus the scope of the search on resource names, titles or abstracts. The e-MIR^2^application allows users to filter results using the concepts within the three classification schemas. There are three combination (combo) boxes—one per schema—containing all the categories shown in Figure 1 in alphabetical order. Users can specify any combination of them, but enter just one category per classification schema at a time.

For the filter over the links, four options are provided: (i) *Indifferent* denotes that user does not mind whether resources have or do not have associated links, (ii)Any indicates that the resources must have one link at least, (iii)Available imposes the requirement that resources must have at least one link working, and finally (iv)Not Available means that resources must have at least one link not working. The option *Indifferent* is checked by default. Users can also filter the searches depending on the origin source from which the information was retrieved. A combo-box shows all the origin sources considered by the system. The last filter is the *Open source* one which restricts retrieval to resources tagged as open source.

Searches are highly customizable. Users can choose any combination of the filters previously described. The search string is not mandatory since a query can be composed by using only filters. If a query is launched without specifying either a search string or filters, then the system returns all the resources contained in the database.

Search results are presented in tabular format. At the top of the table the total number of resources matching the query is shown. Results are displayed in groups of ten items by default. However, the application also allows users to view all results on the same page at one time. The table consists of six columns showing, for each resource, its name, functionalities, type of resource, domains, whether or not the resource is tagged as open source, and a mark to indicate if the resource have available links. In addition, the application presents all the categories associated with each resource. The open source column can only contain two possible values: YES or NO. Finally, the last column may present four different marks: (i) none–when the algorithm does not detect any link to the resource and no links have been manually provided during the manual curation of the resource, (ii) a greenmark–indicating that all links are working,(iii) an orange mark–denoting that some links are not working, and (iv) a red mark-indicating that none of the links are working.

In addition, the resource name is a link itself, which, when followed, provides detailed information regarding the resource. This includes the resource name, whether or not the resource is open source, functionalities, type of resource, domains, links related to the resource and their availability, and information about the paper from which the resource was extracted such as the title, origin source, link to the original source and resource record in XML format.

A typical e-MIR^2^ application scenario would be a researcher who needs to locate databases of diseases with at least one working link. In such a case, the user would select the “Database” category from the “type” combo-box, the “Disease” concept from the “domain” combo-box and finally, select “Any” from the link filter. Other filters and search options would not be relevant in this use case. As shown in Figure 2, the system would return a list of the resources matching the query, one of them tagged as an open source.

## Discussion

Our algorithm scans the titles and abstracts of target manuscripts to identify and extract the information regarding the resources, since pairs for most non open-access medical informatics journals it is not possible to access the full text of articles from bibliographic sources such as PubMed. We believe, however, that in most cases, it is possible to extract the relevant information from the title and/or abstract alone.

To analyze and extract the information, we used a simple method based on pattern-matching techniques. Note that pattern-matching methods are not 100 % accurate and exhaustive. However, according to the results of the evaluation after the manual curation carried out by the experts, they perform well enough to extract the required information from titles and abstracts. Note that other more sophisticated automated methods, or even manual classification —achieving 100 % precision and recall rates—could have been used. However, the performance of these other information extraction methods still depend on the availability of full-text manuscripts —i.e. if the resource name, functionality or domain is not included in the title or abstract, the complete information about the resource will not be detected. We believe that if such information was publicly available it would be worthwhile using more advanced methods for identifying and extracting the relevant information.

Considering the total number of manuscripts analyzed (around 38.000), the rate of identified resources is quite low (around 1.6 %). Based on our own experience in previous works [4], this measure suggests that MI journals traditionally do not publish many papers dealing with informatics resources compared with other journals of disciplines such as BioInformatics. Another possible explanation is that not all journals belonging to the MI field (or at least publishing articles related to the MI field) are not adequately indexed in the ISI Web of Knowledge.

This rate is even smaller if we look at open source resources. Only 24 resources have been identified as open source or using open source technologies. The number of links and open source resources are closely related. Authors provide links usually when they report web applications or freely available resources. Both results suggest that the MIfield has not used—at least, until recently—open tools and standards. The use of open source technologies has become popular in other scientific disciplines, leading to benefits such as resource sharing, lower costs and rapid application development. From the point of view of sharing resources, open source systems and technologies play a key role. In addition, developing resources using open technologies could result in collateral benefits for developing countries, as we have earlier emphasized in the analysis of an international initiative, including partners and experts from Europe, the USA, Latin America and Africa [25]. Applications can be shared for basic research or academic use without strong copyright restrictions. Interoperability among systems could be more reliable and easier. People and institutions from developing countries with scarce funding could also be able to access a wide inventory of resources ranging from clinical applications to educational content. In this regard, it appears that MI still needs to overcome the challenges of introducing open source more ubiquitously for clinical and eHealth applications.

## Conclusions

In this work, we describe the e-MIR^2^ system, a web based application that gives users a means of accessing and adding to existing MI resources. The e-MIR^2^ system architecture has several advantages. These include (i) a search engine that allows users to establish a variety of advanced filters to improve their searches, (ii) a database content that can be automatically updated as new manuscripts are published, or from the suggestions sent by the users, (iii) an extraction engine that can be easily expanded by adding new patterns, and (iv) classification schemas used by the classification engine that have been developed following a novel approach based on data-driven folksonomies.

In the near future, we will be extending the prototype by considering many more MI journals, covering the entire PubMed database, as well as other sources from the biomedical scientific literature. In addition, we are planning to apply and test the same or similar methodologies to other scientific domains.

## Availability and requirements

The e-MIR^2^ web application is freely available at http://www.gib.fi.upm.es/eMIR2. No registration process is required for users to access the application without restrictions.

## Abbreviations

BI, Bioinformatics; e-MIR2, Electronic-Medical Informatics Repository of Resources; ID, Identifier; IJMI, International Journal of Medical Informatics; JAMIA, Journal of the American Medical Informatics Association; JCR, Journal Citation Report; JMIR, Journal of the Medical Internet Research; MI, Medical Informatics.

## Competing interests

The authors declare that they have no competing interests.

## Authors' contributions

GDLC conceived and participated in the design of the study and drafted the manuscript. NNM participated in the design of the study and implemented the system. MGR participated in the design of the study and drafted the manuscript. CK helped to draft and edit the manuscript. VM conceived the study and helped to draft the manuscript. All authors read and approved the final manuscript.

## Pre-publication history

The pre-publication history for this paper can be accessed here:

http://www.biomedcentral.com/1472-6947/12/82/prepub
